# Why have we not yet solved the challenge of plastic degradation by biological means?

**DOI:** 10.1371/journal.pbio.3001979

**Published:** 2023-03-07

**Authors:** Federica Bertocchini, Clemente F. Arias

**Affiliations:** Department of Plant and Microbial Biotechnology, Centro de Investigaciones Biológicas-Margarita Salas, CSIC, Madrid, Spain

## Abstract

The invention of fossil fuel–derived plastics changed and reshaped society for the better; however, their mass production has created an unprecedented accumulation of waste and an environmental crisis. Scientists are searching for better ways to reduce plastic waste than the current methods of mechanical recycling and incineration, which are only partial solutions. Biological means of breaking down plastics have been investigated as alternatives, with studies mostly focusing on using microorganisms to biologically degrade sturdy plastics like polyethylene (PE). Unfortunately, after a few decades of research, biodegradation by microorganisms has not provided the hoped-for results. Recent studies suggest that insects could provide a new avenue for investigation into biotechnological tools, with the discovery of enzymes that can oxidize untreated PE. But how can insects provide a solution that could potentially make a difference? And how can biotechnology revolutionize the plastic industry to stop ongoing/increasing contamination?

## Introduction

Plastics, synthetic molecules designed to be extremely durable, have a polymeric structure made up of tens of thousands of repetitions of small molecules called monomers. Polyethylene (PE) is the most produced among the plastic polymers, accounting for about 30% of total plastic production [1] and, along with polystyrene (PS) and polypropylene (PP) (which together account for 70% of production), is one of the most resistant. This high resistance is at the origin of the plastic waste issue, the accumulation of end-of-life discarded plastic materials and objects. Plastic waste produced since 1950 has surpassed 6 billion tons in total [2], and, globally, a large portion of this waste has ended up in the environment, generating the tremendous plastic pollution issue we face today [1–3].

Most plastic waste management is centered on recycling and incineration, with the alternative being disposal in landfill sites. Mechanical recycling is the most used approach [4], as the technology behind chemical recycling is not yet developed enough to be applied at a large scale. The chemical approach has been reviewed elsewhere [5] and will not be discussed here. Mechanical recycling techniques aim to reutilize plastic building blocks to produce new plastics. However, considering that only a few types of plastics can currently be treated this way, the recycled plastic object is often second rate, and the cycle can be applied only a few times, the contribution of this strategy to removing harmful plastics is ultimately very limited [6]. By contrast, incineration for “energetic revaluation” (as it is called) carries environmental contamination issues [7], which, even in the best-case scenario with state-of-the-art decontaminating filters that only release CO_2_ in the air, should necessarily be avoided.

Plastics that do not enter these disposal pathways end up in the environment directly or indirectly (via landfill sites) [1]. In the environment, after months or years of exposure to factors like light and/or heat, plastic polymers eventually break down through a reaction that involves the formation of reactive radicals, which causes the oxidation of the polymer and, ultimately, the formation of small oxidized compounds (such as ketones, aldehydes, and alcohols) [8–10]. Oxidation (i.e., the introduction of oxygen molecules in the plastic polymer) is a very slow reaction and constitutes a bottleneck in this abiotic degradation chain. Once the long crystalline molecule is broken down into small oxidized fragments, the latter can be utilized (and degraded) by microorganisms such as bacteria or fungi [8].

This raises the question of whether biological means of breaking down plastics could be the solution to the plastic degradation problem. The observation that microorganisms can metabolize the molecules generated by the oxidation of polymers has motivated the search for bacteria and fungi in the environment that are capable of breaking down sturdy fossil fuel–derived plastics [11,12]. Unfortunately, despite being the center of attention in the quest to biologically break down plastics for a few decades, research into biodegradation by microorganisms has not provided clear evidence of degradation. More recently, interest has switched to insects, with larvae of some species of lepidopterans and coleopterans being reported to be able to degrade plastics such as PE and PS [13–17]. Advances of this sort open up new paths to be explored in the quest for a sustainable way to dispose of plastics and point towards research questions that urgently need to be addressed.

In this Unsolved Mystery, we discuss the unanswered questions that permeate the field of plastic degradation by biological means, the solution of which will open a gateway into the plastic waste contamination issue. Why is it so difficult for the bacteria and fungi that have been studied so far to break down sturdy plastics? What might insects provide that could potentially make a difference? And how can biotechnology revolutionize the plastic industry to stop ongoing contamination?

### What makes plastic waste so contaminating?

The presence of plastic debris is becoming a constant panorama in (almost) every region of the planet. The damage created by plastic that becomes entangled within the digestive or respiratory systems of marine or terrestrial creatures is one of the most visible noxious effects of plastic waste. However, the harm caused by plastics is more extensive than that.

Plastics in the environment, landfill sites, and water generate microplastics and, eventually, nanoplastics (collectively known as micro(nano)plastics (MNPs)), small particles that insinuate directly or through the food chain into animal tissues, thereby posing a risk to the health of a whole array of living creatures [18,19]. The contaminating effect of MNPs comes from multiple factors. The first direct effect is the physical accumulation within the digestive tract or filtering system of small animals, causing blockage and damage to the proper functioning of their organs [20]. In addition, MNPs can adsorb pathogens and chemicals, becoming transporters of persistent organic pollutants (POPs) into animal tissues at concentrations higher than those usually found in the environment [21,22]. The consequent effects of these POP-concentrated MNPs is currently a highly prioritized field of research.

Together with the direct physical and indirect chemical influence on the environment, MNPs are themselves a reservoir of dangerous molecules, the plastic additives. Additives are a series of small molecules that are added to the polymer and confer plastics with their resistance and ability to acquire diverse shapes and consistencies. They change the chemical and physical properties of the polymer, making plastic the unique material we know. Several categories of molecules fall under this definition, for example, plasticizers, stabilizers, flame retardants, biocides, and antioxidants [22]. Fragmentation of plastic and formation of MNPs cause these chemical compounds to leach out into their environment, whether that be soil, water, or the digestive system of an animal. Additives are defined as potentially toxic substances [21], and although some studies have described toxic effects for specific molecules [19,20], an exhaustive systematic analysis with a standardized protocol is currently missing. Such a systematic analysis presents intrinsic problems, as plastic manufacturers do not often fully disclose the identity and amounts of additives present in plastic products. Most of the data available so far concern a handful of well-known additives whose toxic effects have been described and confirmed, such as bisphenol A and phthalates [22]. As a consequence, the use of these molecules in plastics has been limited, pointing to the necessity of greater sharing of information and standardized assays to test the potential toxicity of such molecules within plastic polymers.

As a result of these issues, the end target should be the design of plastic polymers with an additive of choice that is nontoxic and easy to degrade. How to reach this point is one of the key questions that needs to be addressed within the plastic industry and the plastic-consuming world economy.

### Where does the field currently stand?

**Fig 1:**
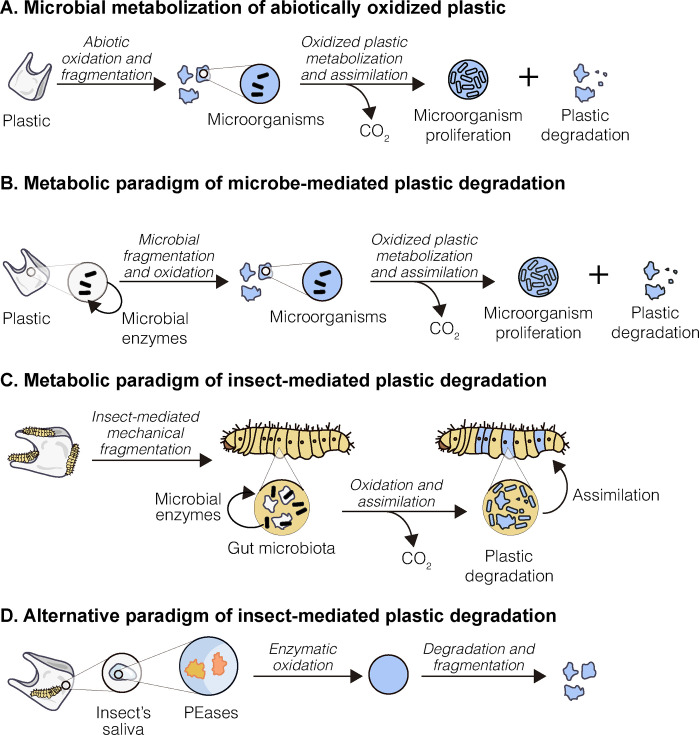
Plastic degradation paradigms. **(A)** The fate of plastics in the environment. Abiotic factors oxidize and fragment plastics. Oxidized plastic (shown in blue) supports the proliferation of microbial populations that can metabolize and assimilate the molecular components of plastics, leading to the release of CO_2_. (**B**) The metabolic paradigm of microbial degradation of plastics. This framework assumes that microorganisms are capable of carrying out the first step of plastic oxidation in the absence of abiotic factors. (**C**) The current view of plastic degradation by insects. The ability of insects to degrade plastic is hypothesized to be mediated by the gut microbiota of the insect. The role of the insect in this scenario would be the mechanical fragmentation of plastics. These small fragments (shown in gray) would then be attacked by microorganisms present in the insect’s gut. The oxidized plastic fragments (shown in blue) would be transformed into molecules that can be assimilated by both the microbiota and the insect’s tissues. (**D**) A new paradigm of insect degradation of plastics. The discovery of the PE-degrading enzymes (PEases) Demetra and Ceres in wax worm saliva evidences an alternative mechanism of insect-mediated plastic degradation, in which the enzymes cause the oxidation and fragmentation of the polymer.

As previously mentioned, once the polymers in plastics have been broken down in the environment by the slow process of oxidation (Fig 1A), the molecules generated can be further degraded by living organisms. The main focus of efforts has been to find microorganisms that can metabolize plastics and use them as a source of carbon and energy, transforming the long polymers into H_2_O and CO_2_ [8,9] (Fig 1B). Much research has gone into microbial degradation of plastics. When microorganisms degrade plastics, the process follows four steps: biodeterioration, biofragmentation, assimilation, and mineralization (see [12] for a review). Microbes capable of assimilating and mineralizing small oxidized molecules derived from oxidation and depolymerization of synthetic polyolefins have been characterized, but the initial two steps are still the most difficult to achieve.

To date, intensive research efforts in the field of biodegradation have resulted in the identification of only a handful of microorganisms that are capable of breaking down these sturdy polymers, with a common major difficulty being overcoming the bottleneck step in plastic degradation, i.e., the oxidation of the polymer [11,12,23–26]. In most cases, fungi and bacteria can only metabolize and fully degrade plastics after the polymers have undergone a preliminary phase of abiotic oxidation (Fig 1A). One apparent exception is represented by poly(ethylene terephthalate) (PET), the polymer disposable bottles are made from. A few microorganisms are in fact capable of breaking it down [27–29]; however, this process is not as straightforward as it seems, and crystalline PET, one of the most used plastics, is not as degradable as it appears to be.

In addition to the fact that effective plastic biodegradation using microorganisms has not yet been achieved, it is crucial to remark that the metabolic use of the polymer as a source of energy entails the release of CO_2_. Therefore, it is in effect a form of slow biologically mediated incineration, an aspect of biodegradation that is usually neglected but should be taken into account.

In addition to microorganisms, some insects have potential as degraders of sturdy polymers. Larvae of the lepidopterans *Plodia interpunctella* and *Galleria mellonella*, and of the coleopterans *Tenebrio molitor* and *Zophobas atrum* have been described as being capable of breaking down PE and/or PS [13–17]. Given the current metabolic paradigm of plastic degradation by biological means, these discoveries were followed by a rush to analyze the gut microbiota of these insects [30–36], a continuing trend (Fig 1C). To date, not only has no specific plastic-degrading microbe been positively identified, but the dependence of plastic degradation on gut bacteria has been called into question [37,38]. So what means do insect larvae use to exert their effect on the most resilient synthetic polymers? Although we do not have the answer to this key question yet, a new discovery in the larva of G. mellonella (the wax worm) is offering an alternative to the metabolic paradigm [39]. Enzymes from the saliva of the larvae are able to break down PE within a few hours of exposure. These enzymes have been identified as phenol-oxidases that can introduce oxygen into the polymer and cause depolymerization (Fig 1D). In this case, plastic is not used to produce energy, and no CO_2_ is released [39]. Oxidation of PE by biological means at room temperature and in aqueous solution represents a new paradigm within plastic degradation, opening up new paths to be explored in the quest for a sustainable way to dispose of plastic residues, and pointing towards unanswered questions that urgently need to be addressed. What are the molecular mechanisms that enable these insects to oxidize PE? How did they evolve? And what is their function in the life cycle of the invertebrate?

### Why do microorganisms have difficulty degrading plastics?

Polymers like PE have a fairly simple chemical structure, with the monomer (-CH_2_-CH_2_-) repeated tens of thousands of times. The extent and spatial arrangement of ramifications within the polymer define the different types of PE (low density, linear low density, or high density). The modification of a lateral group to CH3 or an aromatic ring changes the polymer from PE to PP or PS, respectively (reviewed in [11,40] and references therein).

The sturdy structure of polymers like PE, PS, and PP and their highly hydrophobic nature hinder the colonization of these plastics by many bacteria or fungi, as well as affecting the ability of the microorganisms to grow and use such molecules to satisfy their energetic needs. The access of microorganisms to the plastic molecules can be facilitated by abiotic treatments such as heat or UV light that oxidize the polymer, making it more hydrophilic [11,12,41]. The appearance of smaller compounds after oxidation provides microorganisms with a source of carbon and energy (Fig 1A). Nonetheless, examples have been reported of bacterial species that can grow on untreated plastic. For example, this is the case with various species of *Pseudomonas* (reviewed in [12]), where some degree of plastic degradation and CO_2_ formation can be detected over several months. This is also the case with two bacteria strains isolated from the gut of larvae of *P. interpunctella* (the Indian meal moth), which can grow on PE in a biofilm and break it down within a few weeks [13]. Do these microorganisms synthesize and secrete enzymes capable of nicking the polymer? If that is the case, why does it take several weeks for the bacteria to act? And does this scenario still justify the metabolic paradigm for plastic degradation? No such enzymatic activities have been found for these microorganisms to date. Nevertheless, future research might reveal some still unknown path to plastic degradation by microorganisms.

### Is PET an exception?

PET, which is commonly used to make disposable plastic bottles, accounts for about 9% of total plastic production [42]. Structure-wise, it is a polyester, and as such, it has a less resilient composition than PE (or PP or PS). PET stands out among the other plastic polymers because of the discovery of various enzymes produced by microorganisms that can degrade it. Among the most efficient enzymes that can break down PET are the PET-hydrolyzing enzymes PETase and METase from *Idonella sakienses* [27], and a cutinase from leaf-branch compost named LCC [28]. A culture of *I. sakiensis* can degrade PET very efficiently, with 75% released as CO_2_ in 6 weeks at 30 degrees Celsius, probably utilizing a combination of two aforementioned enzymes. As for the LCC cutinase, the enzyme outperformed other previously described PET-hydrolyzing enzymes in a depolymerization test [28]. However, in both cases, low-crystallinity or amorphous PET was utilized and the degradation capacity of the enzymes drops abruptly when crystalline PET is used instead [43]. A recent discovery added a new polyester hydrolase from plant compost (PHL7), which can highly efficiently degrade amorphous PET, but it shares the same limitations with crystalline PET [29].

In the case of amorphous PET, the molecules are highly accessible to attack by microorganisms, which, combined with the lower hydrophobicity of the polyester, makes the whole scenario quite different from the degradation of crystalline polymers like PE [43,44]. Altogether, this raises questions about the capacity of microorganisms and their enzymatic machinery to work on untreated, commercially discarded, end-of-use plastic polymers: Did enzymes capable of depolymerizing untreated polyolefin-derived plastics, including PET, evolve in nature? And, if so, how would we be able to identify them?

### Can insects provide a new way to dispose of plastics?

The answer to the questions at the end of the previous section may come from studies in insects. As previously mentioned, the larvae of some Lepidoptera and some Coleoptera are able to degrade PE and PS. The first to be described were the caterpillars of the lepidopteran P. interpunctella [13]. In line with the metabolic paradigm of plastic biodegradation, this ability was thought to be attributed to symbiotic microorganisms in the gut of the larvae. A search of the insect gut microbiota resulted in the identification of two bacterial strains, Enterobacter asburiae YT1 and Bacillus sp. YP1, which could grow by forming biofilms on the PE surface, causing consistent damage (i.e., decrease of hydrophobicity, formation of pits and cavities) over a period of 28 days [13]. Similar effects were exerted on PS within the same time frame (28 days) by Exiguobacterium sp. from the gut of mealworms, the larvae of the coleopteran T. molitor [14]. After these pioneering articles, many more followed in the same species or in two other lepidopteran and coleopteran species, G. mellonella and Z. mori, respectively [16,30–35,37,38]. All these larvae can break down PS and/or PE, but how this occurs is still a mystery.

The many years of research in the field of biodegradation by microorganisms weighed heavily on the framework of the experimental approach to plastic degradation by insects. In fact, since the first set of data was gathered, the gut microbiota has been the principal object of investigation (Fig 1C). Despite the considerable volume of data accumulated to date, each dataset on gut microorganisms has pointed to a diverse set of bacterial genera or families [24,30–32,34,36], and the first bacterial species described in P. interpunctella and T. molitor [13] have not been found again [45]. This apparent lack of consistency from studies even on the same species of insect is not surprising if we consider that the digestive apparatus of this type of insects is a remarkably undifferentiated tubular structure that lacks any apparent specialization to harbor a structured microbiome. In the particular case of Lepidoptera, the bacterial communities found in the gut of the larvae are highly heterogenous and seem to be determined by the specific conditions in which each individual lives [46].

The role of the larval gut microbiota in plastic degradation has also been called into question by the suggestion that the animal itself might have evolved some kind of machinery for this function [37,38]. Insights in this direction came from recent data on the larvae of G. mellonella. These invertebrates had already shown a remarkably rapid ability to oxidize untreated PE (within hours of contact) [39], but the modality and origin of this action was unknown. Until now. Wax worms have been shown to degrade PE via the action of their saliva. Within hours of exposure in aqueous solution and at room temperature, the saliva oxidizes the polymer leading to the formation of small oxidized molecules as by-products of degradation [47]. The same study revealed the presence of wax worm enzymes in the larvae saliva belonging to the family of phenol oxidase activities, called PEases [47] (Fig 1D). This was the first report of enzymes being able to break down PE and make the polymer more accessible to enzymatic attack without any abiotic pretreatment.

**Fig 2:**
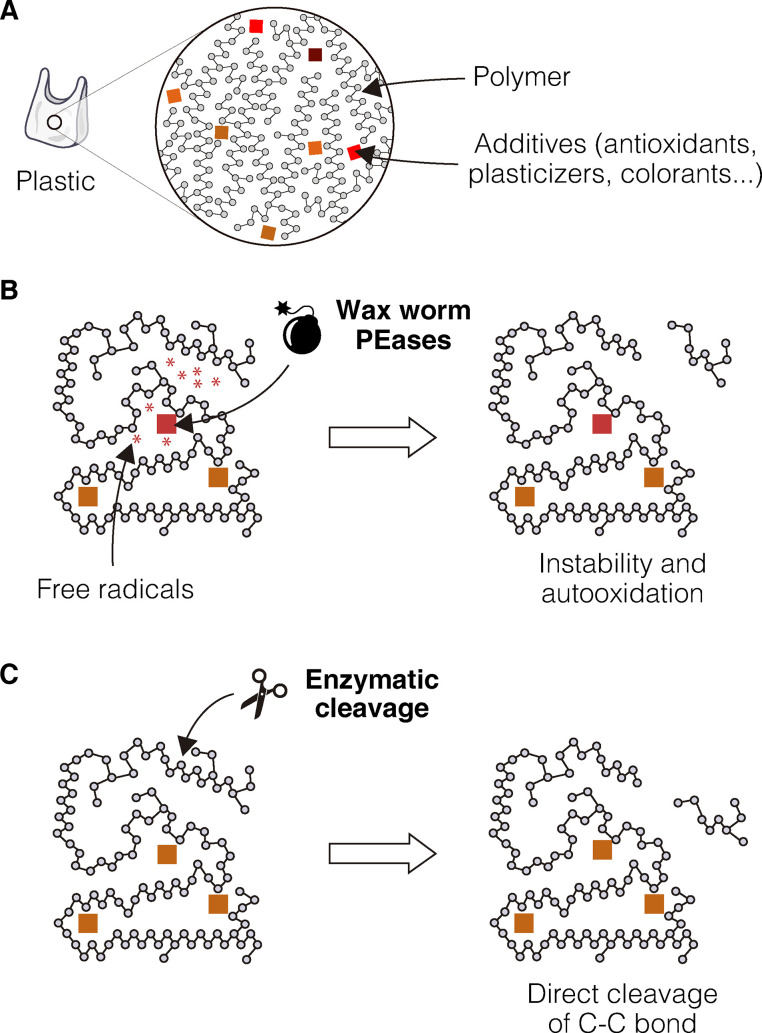
Alternative hypothesis for plastic degradation by biological means. **(A)** Polyethylene (PE), showing the polymer and the additives (colored squares) that form the PE. (**B**) A newly proposed mechanism, whereby plastic is broken down by PEase-mediated free radical formation, leading to an autooxidative chain reaction. (**C**) Classical view of plastic degradation via a direct enzymatic cut on the C-C chain.

One immediate question that arises is what is the molecular mechanism driving this enzymatic oxidation? To answer this question, the issue of the role of phenol oxidases in Lepidoptera saliva must first be addressed: What are they doing there? Phenolic compounds are present in large quantities in plants and represent a defense against the attack of caterpillars and other herbivorous insects [48]. The capacity to neutralize phenolic compounds allow the larvae to feed on leaves or other plant derivatives that contain these molecules (pollen, resins, etc.). Therefore, these aromatic compounds could be the original target for the two PE-degrading wax worm enzymes. But how can they act on PE? In abiotic PE degradation, the key step triggered by abiotic factors (light, UV, etc.) is the formation of free radicals. A chain reaction known as autooxidation then follows that oxidizes the polymer and breaks it into small compounds like alcohols, ketones, and aldehydes [10]. Could a similar mechanism be responsible for oxidation by the wax worm enzymes? If that were the case, a source of free radicals would be necessary for the reaction to occur. Some plastic additives bear similarities to the plant phenolic compounds and could indeed become targets of the wax worm phenol oxidases, leading to the formation of free radicals and, ultimately, to the oxidation of the polymer (Fig 2A and 2B). However, this is only a hypothesis and other scenarios might occur. For example, the chemical similarities between wax and PE cannot be ignored: In some still obscure fashion, the larvae might recognize PE as if it were wax. This would point to an obvious alternative mechanism of plastic degradation—a direct attack and oxidation of the aliphatic chain (Fig 2C). In this line of thought, one of the ongoing hypotheses for how an aliphatic chain might be oxidized and broken is the end-of-chain oxidation by abstraction of monomers [49]. This is comparable to the shortening of short aliphatic chains by microbial alkane hydroxylases [50]. To date, the oxidation and depolymerization mechanisms still remain an unsolved mystery.

The two newly identified PEases, Demetra and Ceres, are secreted by the larvae and oxidize the polymer outside the insect’s body [47]. Could this mechanism rule out the metabolic paradigm of plastic degradation? By oxidizing the polymer and causing the formation of small oxidizing molecules, the two enzymes could potentially be preparing food for the gut microbiota; if this proves to be true, then the plastics could still be metabolized by gut microorganisms, just not directly, meaning that no specific bacteria or fungi would be required to do the job. As previously noted, even if lepidopteran larvae harbor microbial communities in their guts, the structure and composition of these communities is highly heterogeneous among individuals of the same species; in fact, it seems to be determined by the particular conditions in which each individual lives [46]. A change of the microbiota as a consequence of a plastic-based diet, with enrichment of certain bacteria genera, does not necessarily mean that the enriched populations are capable to degrade PE or PS. Assuming that only one bacterial species exists with this ability is just as speculative as assuming that many species exist that will do the job. However, most reports pointing to G. mellonella microbiota as the culprit for PE and/or PS degradation have assumed this postulate and search for potential bacterial candidates analyzing the changes in the microbiome after exposure to a PE- or PS-rich diet [31,32,34,51–53]. This approach has yielded no specific wax worm plastic-degrading bacterial species capable of growing in isolation on plastics, or any proof of bacteria-specific degradation. But once plastics are oxidized and depolymerized by the saliva enzymes, the small oxidized molecules could very well be the object of attention of any of the species present in the gut microbiota. This reasoning does not rule out the possibility of microbial specialization in the wax worm gut, but this is not reflected in the data about the wax worm microbiota and plastic degradation. The presence of enzymes within insect saliva is hardly new [54,55], but their function has been related mostly to defense against parasites or the detoxification of chemicals that could jeopardize the well-being of the larva. This scenario seems to suggest a predigestive role for secreted enzymes; however, we cannot exclude any possibility at this point, and only with an in-depth study of the enzymes, both structurally and biochemically, and a better understanding of their evolutionary history will we be able to discern their real function within the animal, and their whole array of potential uses.

## Conclusion

### The path to the future

Degradation of fossil fuel–derived plastics by biological means has been offered as a potential solution to the current plastic waste contamination emergency. The “biological means” have always been presumed to be bacteria or fungi that could carry out the longed-for plastic biodegradation within the environment. However, despite the huge amount of work that has been devoted to this issue, no solution has yet emerged from that direction. Only a handful of microorganisms seem able to affect sturdy polymers such as PE without an abiotic pretreatment, and, even in this case, it takes a few weeks for the effect to appear. Moreover, in these few cases, no microorganism-derived enzymatic activities have yet been described. Leaving bacteria and fungi aside, insects now seem to offer hope within the field of plastic bioremediation. The Lepidoptera G. mellonella can use its saliva to oxidize PE within a few hours following exposure via the action of phenol oxidases. The discovery of such insect PEases has begun to unravel the mystery of how insect larvae can degrade PE and, probably, PS (i.e., using larvae’s enzymes in the saliva), but has opened up a Pandora’s box of new unsolved mysteries. How do these enzymes work, molecularly? Do they have superficial catalytic sites? How do they act on the sturdy plastic structure? What is their function in nature? Do both enzymes described so far work in the same way, or do they complement each other? Do other enzymes of the same type exist in nature? How did they evolve?

The sooner we can start answering this array of questions, the faster we will be able to reshape the global plastic economy by developing a cutting-edge biotechnological tool for a controlled disposal of plastic waste, reutilizing the by-products of degradation in an upcycling modality, rethinking the formulae of plastic polymers taking into account what part of the plastic is subject to the biological degradation, and making it prone to depolymerization by the chosen enzymes.

## Abbreviations

MNP: micro(nano)plastic
PE: polyethylene
PET: poly(ethylene terephthalate)
POP: persistent organic pollutant
PP: polypropylene
PS: polystyrene
